# Clinical and laboratory features of seven patients with acute myeloid leukemia (AML)-M2/M3 and elevated myeloblasts and abnormal promyelocytes

**DOI:** 10.1186/s12935-014-0111-y

**Published:** 2014-12-31

**Authors:** GanLin He, ChunYan Wang, QingEn Li, Huo Tan, FuXiong Chen, ZhenQian Huang, BaoDan Yu, LiXia Zheng, RunHui Zheng, Dan Liu

**Affiliations:** Department of Hematology, The First Affiliated Hospital of Guangzhou Medical University, No. 1 Kangda Road, Haizhu District, 510000 Guangzhou, China; Department of Pediatrics, The First Affiliated Hospital of Guangzhou Medical University, Kangda Road, Guangzhou, China; State Key Laboratory of Respiratory Disease, The First Affiliated Hospital of Guangzhou Medical University, Kangda Road, Guangzhou, China

**Keywords:** Acute myeloid leukemia (AML), AML-M2/M3 subtype, Mixed phenotype, Myeloblasts, Abnormal promyelocytes

## Abstract

**Background:**

There is limited information on a special subtype of Acute myeloid leukemia (AML) characterized by >20% myeloblasts and >20% abnormal promyelocytes in bone marrow and peripheral blood.

**Objective:**

The objective of the present investigation was to explore the clinical and laboratory features of seven patients with AML-M2/M3.

**Method:**

We retrospectively assessed cell morphology, cytochemistry, immunophenotype, cytogenetics, and clinical features of seven patients with this rare subtype of AML.

**Results:**

All seven cases had thrombocytopenia, coagulation abnormalities, >20% myeloblasts and abnormal promyelocytes. The PML/RARα fusion gene was present in six patients and two patients presented a mixed PML/RARα and AML1/ETO genotype. Five cases achieved CR and two cases did not achieve remission and one case transform into AML-M2 after CR_1_.

**Conclusions:**

The clinical and laboratory features of seven patients with AML-M2/M3 are demonstrated in the present study, providing information on the FAB sub-classification.

## Background

Acute myeloid leukemia (AML) is characterized by clonal expansion of myeloid blasts in bone marrow (BM), peripheral blood (PB), or other tissues. In the World Health Organization (WHO) scheme (2008), a myeloid neoplasm with 20% or more myeloblasts in the PB or BM is considered to be AML. Acute myeloid leukemia with the gene rearrangement t (8; 21) (q22; q22); (AML1/ETO), which is also referred to as the RUNX1 gene, and AML with the rearrangement t (15; 17) (q22; q12) (PML/RARα) are considered distinct AML subtypes. In the French-American-British (FAB) classification scheme for AML, the criteria for AML-M2 is 30% to 89% myeloblasts, >10% promyelocytes and neutrophilic myelocyte, and <20% monocytes [1-9].

Two cases of AML with simultaneous PML/RARa and AML1/ETO gene rearrangement have been reported [10]. We have encountered seven cases with myeloblast fractions ranging from 36 − 85% and a fraction of abnormal promyelocytes from 24 − 49.5%, with one patient presenting with simultaneous AML1/ETO and PML/RARα fusion genes [11-18]. In this paper, we retrospectively analyze all seven cases diagnosed as AML-M2/M3, to present a general picture of the clinical symptoms, laboratory results, prognosis, and treatment responses of this unusual AML subtype.

## Materials and methods

### Clinical information

The current study was approved by the Department of Hematology of the First Affiliated Hospital of Guangzhou Medical University. Patients were enrolled and treated from Jan. 2008 to Jun. 2012. Study eligibility criteria included availability of bone marrow histology and cytogenetic information at the time of referral to the Department of Hematology. Acute myeloid leukemia was diagnosed according to the World Health Organization criteria [1,2,8]. During the study period, seven patients, aged 8 to 76, were newly diagnosed with AML-M2/M3 based on >30% myeloblasts and >20% abnormal promyelocytes as well as predominant myeloperoxidase (MPO or POX) and naphthol AS-D chloroacetate esterase (AS-DCE, or specific esterase, SE) positive status. Bone marrow smears, PB smears, and detailed morphological, immunochemical, and cytogenetic analyses of BM biopsy tissue were conducted for each patient. The myeloid lineage was assessed using antibodies against CD9, CD11b, CD13, CD15, CD33, CD34, CD38, CD45, CD56, CD64, CD117, HLA-DR, and cMPO. The lymphocyte T cell lineage was assessed using antibodies against CD2, CD3, CD5 and CD7, while the B cell lineage was assessed using antibodies against CD19, CD20, CD22, and CD79a.

### Flow cytometry

The flow cytometer of COULTER EPICS XL type with 488 nm excitation light source was used in the present investigation, and System II software was employed for analysis. After calibration with Fluorescent Microspheres and adjustment Fluorescence compensation of the machine, three-color fluorescent staining of the same type sample was done to serve as negative control to exclude nonspecific fluorescence staining. According to the degree of expression of CD45 and particle size of SSC, the cells were divided into granulocytes, monocytes, lymphocytes, immature lymphocyte populations and immature myeloid cells group, red blood cells and debris cells. After analysis of each cell group, naive cells were determined. The expression of antigen was analyzed by two-dimensional phenotypic spectrum.

### Case studies

#### Case 1: A 10-year-old male presented with fever

**Figure 1:**
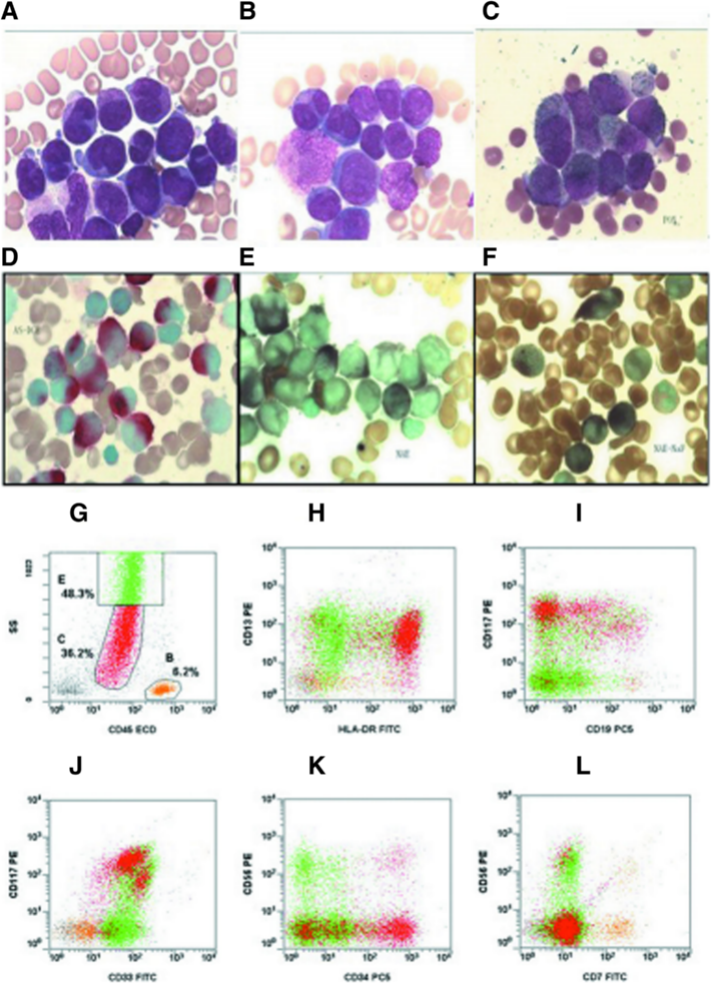
**Morphological and immunological characteristics of BM cells in AML-M2/M3 patients. A**, **B**: Bone marrow smears demonstrated hyperplasia of the myeloid lineage. Myeloblasts and abnormal promyelocytes accounted for >30% of BM cells. Abnormal promyelocytes were round, oval, or elliptical. The cytoplasm contained intense dust-like azurophilic granulation and some abnormal promyelocytes contained Auer rods. The nuclear size and shape were irregular; the nucleus could either appear kidney-shape, cerebriform, or even bi-lobed, with inconspicuous to prominent nucleoli. **C**: MPO: 99% positive; **D**: AS-DCE: 70% positive; **E**: NSE: particle positive; **F**: NSE positive cells were inhibited by NaF. **G-L**: Immunophenotype as determined by FCM for Case 7 showing two distinct groups of blast cells (red and green).

Bone marrow smears showed hyperactivity of the myeloid lineage; myeloblasts and abnormal promyelocytes together comprised 100% of the BM cells. Abnormal promyelocytes, but with no Auer rods, accounted for 38% of all cells. Some myeloblasts exhibited one or two prominent nucleoli. Results of immunohistochemistry of BM biopsy tissue were consistent with AML: MPO(+++), CD117(+++), TdT(-), CD34(-), L26(-), UCHL-1(+),CD3(-), CD79a partial(+), Fe(-), and reticular fiber(-) (Figure 1).

#### Case 2: A 57-year-old male presented with vertigo, pale and fever

Bone marrow smears revealed hyperactivity of the myeloid lineage. Results of a BM biopsy were consistent with AML. Most AML cells were MPO (+), CD20 partial (+), CD79а partial (+), CD3 partial (+), some CD138 (+), CD4 (-), CD5 (-), Ag (+), and Fe (-).

#### Case 3: A 57-year-old female presented with mucocutaneous hemorrhage and fever

In BM smears, the myeloid lineage was hyperactive, with 39.5% myeloblasts and 49% abnormal promyelocytes.

#### Case 4: A 32-year-old male presented with fever and mucocutaneous hemorrhage

Results of BM biopsy were consistent with AML: MPO(+), CD117(+), LCA(+), Ki-67 approximately 20%(+), CD3(-), CD34(-),TdT(-), CK(-), Ag(++), and Fe(-).

#### Cases 5: A 76-year-old male presented with dizziness

**Figure 2:**
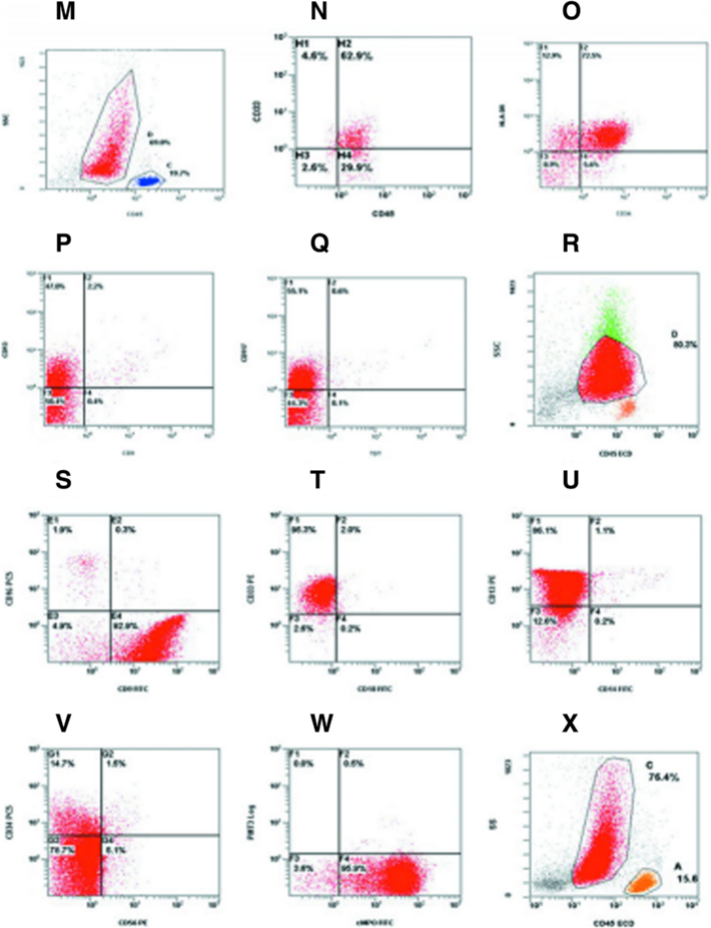
**FCM of AML-M2/M3 patients. M-Q**: Immunophenotype of Case 2 as measured by FCM. **R-W**: Immunophenotype of Case 3. **X**: Immunophenotype of Case 5. Bone marrow karyocytes contained a group of abnormal cells (group C, red) accounting for 76.4% of the total.

Bone marrow smears revealed 42.5% myeloblasts and 30% abnormal promyelocytes. Cytochemistry indicated 99% MPO (+) cells. Most cells also appeared AS-DCE (+). The immunophenotype was MPO (+), CD117 (+), LCA (+), about 20% Ki-67(+), CD3 (-), CD34 (-), TdT (-), CK (-), Ag (++), and Fe (-). Cytogenetic analysis indicated the presence of the PML/RARа fusion gene, S type. Immunophenotyping by FCM revealed that 76.4% of BM karyocytes were abnormal (group C, red). Most cells were weakly CD45 (+), while 30.9% were CD13 (+) 11.6% CD15 (+), 95.5% CD33 (+), 96.5% CD56 (+), 16.9% CD64 (+), 78.4% CD117 (+), and 84.3% were positive for the cytoplasmic antigen of cMPO. Cells were negative for antigens of the lymphocytic lineage (Figure 2).

#### Case 6: An 8-year-old male presented with rashes

Bone marrow smears revealed 17% myeloblasts and 49.5% abnormal promyelocytes. Peripheral blood smears consisted of 36% myeloblasts and 28% promyelocytes. Cells were positive for the PML/RARа fusion gene, S type, and the AML1/ETO fusion gene. The immunophenotype by FCM was approximately 44.9% bone marrow blast cells. Within this population, 29.6% were CD34/HLA-DR (+), 6.5% CD15 (+), 0.2% CD14/CD11b (+), and 3.5% HLA-DR/CD13 (+). No antigens of the lymphocytic lineage were detected.

#### Case 7: A 30-year-old female presented with bone pain and fever

**Figure 3:**
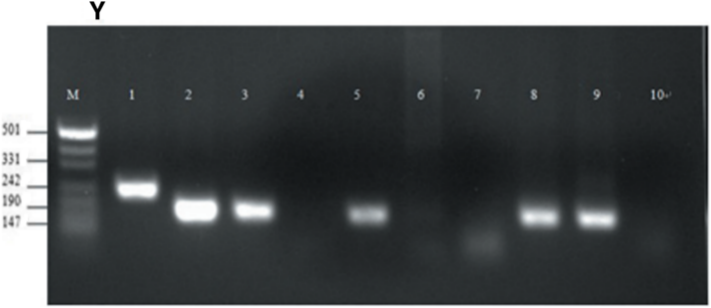
**Y:** **Nested RT-PCR for Case 7: M: marker.** 1 − 7: PML/RARа fusion gene. 1: mutation, type S positive control; 2: patient type S PCR products; 3: type S positive control; 4: healthy control, type S PCR products; 5: type L positive control; 6: patient type L PCR products; 7: healthy control, type L PCR products. 8-10: AML1/ETO fusion gene; 8: positive control; 9: patient PCR products; 10: healthy control PCR products.

Bone marrow smears revealed 49% myeloblasts and 13% abnormal promyelocytes, while the cells in the PB smear were 14% myeloblasts and 13% promyelocytes. The BM cells harbored both the PML/RARа fusion gene, S type, and the AML1/ETO fusion gene. Immunophenotyping by FCM revealed two groups of blast cells. The SSC lower cell group was approximately 44.9% of the total and this population was 13.5% CD11b(+), 90.8% CD13(+), 40.3% CD15(+), 82.6% CD33(+), 62.3% CD34(+), 12.4% CD56(+), 40.3% CD64(+), 88.0% CD117(+), 85.9% HLA-DR (+), and 97.8% cMPO(+), while no antigens of other lineages were expressed. The SSC lower cell group was approximately 48.3% of the total and this population was 46.6% CD11b(+), 94.9% CD13(+), 17.3% CD14(+), 95.3% CD15(+), 83.0% CD33(+), 26.1%(+) CD56, 70.4% CD64(+), 43.8% CD117(+), 35.0% HLA-DR(+), and 98.9% cMPO(+). Antigens of other lineages were not expressed (Figure 3).

Patients’ clinical profiles, the proportions of myeloblasts and abnormal promyelocytes in BM and PB smears, immunocytochemical determination of MPO(+) and AS-DCE(+) fractions, results of immunophenotyping by flow cytometry, and results of cytogenetic analysis for all seven case patients are summarized in Tables 1, 2, 3, and 4. The majority of cells were positive for antigens of the myeloid lineage and negative for antigens of the lymphocytic lineage.

**Table 1:** **AML-M2/M3 case profiles**

**Variable**	**Case 1**	**Case 2**	**Case 3**	**Case 4**	**Case 5**	**Case 6**	**Case 7**	**%(7/7)**
**Aex/age**	**Male/10**	**Male/57**	**Female/57**	**Male/32**	**Male/76**	**Male/8**	**Female/30**	
Fever	38.5°C	37.8°C	37.8°C	38°C	36.8°C	38.4°C	38°C	85.7 (6/7)
Sternum pressing pain	+	±	+	+	-	+	+	71.4 (5/7)
Hepatomegaly or splenomegaly	+	-	+	-	-	-	-	28.6 (2/7)
Hemorrhagic symptoms	+	-	+	+	+	-	+	71.4 (5/7)

+, positive, showed the symptoms; -, negative, no such symptom; ±, the symptoms were not prominent.

**Table 2:** **Laboratory features of 7 patients with AML-M2/M3**

**Variable**	**Case 1**	**Case 2**	**Case 3**	**Case 4**	**Case 5**	**Case 6**	**Case 7**	**Higher than**	**Lower than**
								**Normal, n (%)**	**Normal, n(%)**
WBC (×10^9^/L)	112	0.8	119.1	60.9	1.5	17.8	10.1	5(71.4)	2(28.6)
RBC (×10^12^/L)	4.1	1.6	3.3	2.6	2.2	3.8	3.56	0(0.0)	2(28.6)
HB (g/L)	122	57	93	82	77	105	119	0(0.0)	6(85.7)
Platelets (×10^9^/L)	75	18	18	26	52	65	39	0(0.0)	7(100)
LDH (U/L)	294	192	706	383	364	937	1037	6(85.7)	1(14.6)
PT (sec)	15.8	15.1	25.2	15.9	13.5	13.3	13.6	4(57.1)	0(0.0)
Fibrinogen (g/L)	1.4	5.1	0.6	2	3.6	3.1	4.23	2(28.6)	2(28.6)
D-dimer (ng/ml)	>10000	1249	3607	3310	821	-	881	6(85.7)	0(0.0)
BM Myeloblasts	62%	47.50%	39.50%	85%	42.50%	17%	49%	7(100)	0(0.0)
BM abnormal promyelocytes	38%	35.50%	49.50%	11.50%	30%	49.50%	29%	7(100)	0(0.0)
PB Myeloblasts	61%	0	47%	67%	1%	36%	14%	5(71.4)	0(0.0)
PB abnormal promyelocytes	17%	0	50%	24%	0	28%	13%	5(71.4)	0(0.0)
MPO of BM smears	62% strongly positive	94% strongly positive	99% positive	>90% strongly positive	99% positive	-	95% positive		
AS-DCE of BM smears	48% positive	23% positive	70% positive	19% positive	>90% positive	-	50% positive		
NSE of BM smears	-	8% weakly positive	<20% positive	>90% strongly positive	-	-	-		

WBC, white blood cell count in peripheral blood; RBC, red blood cell count; HB, hemoglobin; LDH, lactate dehydrogenase; PT, prothrombin time; APTT, activated partial thromboplastin time; BM, bone marrow; PB, peripheral blood; MPO, myeloperoxidase; AS-DCE, naphthol AS-D chloroacetate esterase; NSE, non-specific esterase (or alpha-naphthol acetate esterase, a-NAE); -, no results available.

**Table 3:** **Immunophenotype of 7 patients with AML-M2/M3 by FCM**

**No.**	**CD45(blast)**		**CD9**	**CD13**	**CD33**	**CD34**	**cMPO**	**CD117**	**HLA-DR**	**CD11b**	**CD15**	**CD38**	**CD56**	**D64**
Case 1	81.1		47.5	32.7	46.9	―	―	―	―	―	―	41.0	―	―
Case 2	69.0		―	47.0	62.9	78.1	―	55.1	85.4	―	―	―	―	―
Case 3	80.3		93.2	87.2	97.3	16.2	96.4	―	―	―	―	―	―	―
Case 4	83.7		92.5	―	97.0	―	98.9	16.4	―	12.7	―	―	―	86.7
Case 5	76.4		―	30.9	95.5	―	78.4	78.4	―	―	11.6	―	96.5	16.9
Case 6	44.9		CD34/HLA-DR 29.6%, CD15 6.5%, CD14/CD11b 0.2%, HLA-DR/CD13 3.5%											
Case 7	84.5	36.2	―	90.8	82.6	62.3	97.8	88.0	85.9	13.5	40.3	―	12.4	7.9
		48.3	―	94.9	83.0	―	98.9	43.8	35.0	46.6	95.3	―	26.1	70.4

―, these antibodies were expressed in less than 10% of cells.

**Table 4:** **Genetics features of 7 patients with AML-M2/M3**

**Variable**								**Total**	
	**Case 1**	**Case 2**	**Case 3**	**Case 4**	**Case 5**	**Case 6**	**Case7**	**+**	**-**
PML-RARa	+/S	-	+/S	+/S&L	+/S	+/S	+/S	5	1
ETO	non	non	non	non	non	+	+	2	0
BCR-ABL	-	+	-	non	non	-	non	1	3

S, short type of PML-RARa; L, long type of PML-RARa; non, not examined.

### Chemotherapy

Five of the six cases were treated by standard chemotherapy [17,18] (daunorubicin + cytarabine, DA) combined with all-trans retinoic acid (ATRA) or arsenic trioxide (ATO). The other case (case 6) was first treated with ATRA, compound realgar, and natural indigo tablets (CRINT), and then given reduced dose DA [19]. After the initial treatment, 5 cases achieved complete remission (CR), including one case with recurrence after three months that transformed into AML-M2 (Case 6). The average time to achieve CR was 37 days. Two cases did not achieved remission (including one death). After stem cell transplantation, patients with AML-M2/M3 might have prolonged event-free survival (Table 5).

**Table 5:** **Follow-up of 7 patients with AML-M2/M3 until 30th june, 2012**

	**Chemotherapy treatment**	**Subsequent**	Time*(days, d)	**Final diagnosis**
	**In our deparmett**	**Treatment**		
Case 1	3 cycles	allo-SCT**	1630d	CR
Case 2	4 cycles	auto-SCT	1492d	CR
Case 3	1 cycle	-	3d	Death
Case 4	5 cycles	allo-SCT	766d	CR
Case 5	1 cycle	-	>29d	Non-remission, lost at follow-up
Case 6	4 cycles	-	467d	Recurrence, transformed into AMLM2
Case 7	1 cycle	-	53d	CR

allo-SCT, allogeneic stem cell transplantation; auto-SCT: autologous stem cell transplantation.

*closing date: 30 June, 2012.

^**^the patient had the allo-SCT in another hospital.

## Results

When newly diagnosis, 85.7% of case patients (6/7) had fever, 71.4% (5/7) had sternum pressing pain, hemorrhagic symptoms, and anemia, 28.6% (2/7) presented with hepatomegaly or splenomegaly, 100% had leukocytosis or leukopenia, and thrombocytopenia, 28.6% showed reduced hemocytes (all lineages), 100% had coagulation abnormalities, and 85.7% had elevated serum LDH. All patients had >20% myeloblasts and >20% abnormal promyelocytes, of which 71.4% had >30% BM myeloblasts and abnormal promyelocytes. All six patients tested were MPO (+). Six patients were PML/RARа positive and two of these patients harbored both the AML1/ETO and PML/RARа fusion genes. Immunophenotyping (Table 3) showed that BM and PB cells expressed antigens of the myeloid lineage, including CD9, CD11b, CD13, CD15, CD33, CD34, CD38, CD45, CD56, CD64, CD117, HLA-DR, and cMPO.

## Discussion

The FAB classification scheme (1976) divided AML into subtypes based on the morphology of BM or PB cells, while the current World Health Organization (WHO) classification system incorporates morphology, cytogenetics, molecular genetics, and immunological markers for the diagnosis of AML subtypes [2,5,8]. Acute myeloid leukemia is divided into two subtypes with different treatments and prognoses, AML-M2 and APL (AML-M3), depending on the magnitude of the increase in myeloblasts or abnormal promyelocytes [3-5]. Here, we provide evidence for a mixed phenotype (AML-M2/M3) with the characteristics of both AML-M2 and AML-M3. The clonal and temporal relationship between APL and the secondary AML subtype are usually obscure [20-23]. In fact, a chimeric M3:M2 case of acute promyelocytic leukemia was recently presented [24].

According to the diagnostic criteria for AML, AML-M2 is characterized by significantly increased myeloblasts, while AML-M3 is associated with abnormal promyelocytes. However, six of our cases meet the diagnostic criteria for both AML-M2 (>30% myeloblasts) and AML-M3 (>30% abnormal promyelocytes). We also observed two patients with both the AML1/ETO and the PML/RARα fusion gene. Such cases cannot be classified as AML-M2 or AML-M3, so we suggest a new subtype of AML, AML-M2/M3 [2,5,8,15-17].

The diagnosis of AML-M2/M3 requires all available information, including morphology, cytochemistry, immunophenotyping, genetics, and clinical features [2,8]. In particular, BM cell morphology and the presence of the PML/RARа and/or AML1/ETO fusion gene define this subtype, while the immunophenotype is not sufficiently distinct from previously defined subtypes for diagnosis.

In BM and PB smears, all cases showed a simultaneous increase in myeloblasts and abnormal promyelocytes. Some promyelocytes contained dust-like or sparsely distributed granules. Promyelocyte nuclei were kidney-shape or even bi-lobed with inconspicuous to prominent nucleoli. Some promyelocytes also contained Aure rods. These morphological characteristics are distinct from the typical abnormal promyelocytic cells observed in AML-M3 [1-3].

Immunophenotyping showed that the SSCs expressed CD9, CD11b, CD13, CD15, CD33, CD34, CD38, CD45, CD56, CD64, CD117, and HLA-DR, antigens characteristic of both AML-M2 and AML-M3. Three cases expressed BM cells strongly positive for CD9 and four cases exhibited cells strongly positive for CD13 and CD33. Two of 6 cases exhibited cells with CD117 expression. In addition, CD34, cMPO, CD117, HLA-DR, CD11b, CD15, CD38, CD56, and CD64 were expressed in some cases. However, it is difficult to distinguished AML-M2 from AML-M3 according to immunophenotype [25-27].

It is uncertain whether the two cases with both fusion genes harbor two genetic rearrangements within the same clone or two independent clones with different rearrangements. Unfortunately, as this was a retrospective study, no further analysis was possible and there was no information on how the various FACS and molecular assays were performed. Further studies are needed to examine the frequency of this double genetic rearrangement in AML-M2/M3.

Many of the clinical characteristics of AML-M2/M3 are common to other subtypes of leukemia, including anemia, fever, hemorrhage, and infiltration. However, this group of patients was prone to DIC, cerebral hemorrhage, and other serious complications, with high mortality rates. Moreover, patients showed a trend for recurrence and transformation into AML-M2 [3,4]. Although some clinical procedures including transplantation of stem cell has been proposed in the present investigation, the suggestion was preliminary, not original and might not be general for all similar cases. Anyhow, we analyzed the treatment effects of ATRA, ATO, or CRINT combined with the DA regimen on seven patients with AML-M2/M3, whose clinical and laboratory features were analyzed scientifically and the data revealed that there were elevated myeloblasts and abnormal promyelocytes. Because up to now, there has been no uncontested criterion for the FAB sub-classification of AML, analyzing particular cases of AML without scientific genaralization of considered particular cases still is of some significance. Thus, it remains to be investigated in FAB sub-classification of AML and its clinical treatment. In addition, further studies are needed to examine the frequency of this double genetic rearrangement in AML-M2/M3.

At present, there are no guidelines for the treatment of this new subtype of AML. All of the patients were treated with ATRA, ATO, or CRINT combined with the DA regimen and five achieved complete temporary remission. Our initial observations suggest that hematopoietic stem cell transplantation may be beneficial for long-term survival. Therefore, the significance of the present study was that the patients with AML could be treated with ATRA, ATO, or CRINT combined with the DA regimen, and hematopoietic stem cell transplantation might be beneficial for long-term survival, possibly providing data for the guidelines for the FAB sub-classification and treatment of this new subtype of AML.

In the present study, we identified a novel entity of AML according to FAB classification and examined its clinical characteristics. Although the “gray-zone” entity of each of the FAB sub-classification has been recognized since the first proposal of FAB classification in 1976, unfortunately, this issue was not endowed with clear solution. Later, in a “proposed revised criteria for the classification of AML” by the FAB cooperative group in 1985 [28], the potential fermentation concerning discrimination between M2 and M3 has been asserted. The group noted that M2 was diagnosed when mature myeloid cells were over 10%, even though “few” promyelocytes or later cells were present, indicating that M2 does not contain cases with “massive” promyelocytes. Therefore, according to the revised FAB criteria, the presented cases were not totally consistent with M2. In contrast, blasts percentage >30% did not exclude M3 diagnosis. Indeed, myeloblasts occupy >30% in about 10% of M3 cases [29]. Microscopic features of promyelocytes in the presented cases including sparse granules and bilobed nucleus were characteristics of AML M3 variant. Thus, we regarded the cases as AML-M2/M3, which remains to be further explored. Anyhow, the present investigation added new information for the analysis of AML-M2/M3.

In conclusion, the clinical and laboratory features of seven patients with AML-M2/M3 are demonstrated in the present study, providing information on the FAB sub-classification.

## References

[1] Jaffe ES, Harris NL, Stein H, Vardiman JW (2001). World Health Organization Classification of Tumours. Pathology and Genetics of Tumours of Hematopoietic and Lymphoid Tissues.

[2] Swerdlow SH, Campo E, Harris NL, Jaffe ES, Pileri SA, Stein H, Thiele J, Vardiman JW (2008). WHO Classification of Tumours of Hematopoietic and Lymphoid Tissues.

[3] Estey E, Dohner H (2006). Acute myeloid leukemia. Lancet.

[4] Lowenberg B, Downing JR, Burnett A (1999). Acute myeloid leukemia. N Engl J Med.

[5] Bennett JM, Catovsky D, Daniel MT, Flandrin G, Galton DA, Gralnick HR, Sultan C (1985). Proposed revised criteria for the classification of acute myeloid leukemia. A report of the French-American-British Cooperative Group. Ann Intern Med.

[6] De Jonge HJ, Huls G, de Bont ES (2011). Gene expression profiling in acute myeloid leukemia. Neth J of Med.

[7] Vardiman JW, Harris NL, Brunning RD (2002). The World Health Organization (WHO) classification of the myeloid neoplasms. Blood.

[8] Vardiman JW, Thiele J, Arber DA, Brunning RD, Borowitz MJ, Porwit A, Harris NL, Le Beau MM, Hellström-Lindberg E, Tefferi A, Bloomfield CD (2009). The 2008 revision of the World Health Organization (WHO) classification of myeloid neoplasms and acute leukemia: rationale and important changes. Blood.

[9] Kelly LM, Gilliland DG (2002). Genetics of myeloid leukemia. Annu Rev Genomics Hum Genet.

[10] Stavroyianni N, Kalmantis T, Yataganas X (1999). Simultaneous PML/RAR alpha and AML 1/ETO gene rearrangements in a patient with acute myeloid leukemia. Leukemia.

[11] Schlenk RF, Dohner K, Krauter J, Fröhling S, Corbacioglu A, Bullinger L, Habdank M, Späth D, Morgan M, Benner A, Schlegelberger B, Heil G, Ganser A, Döhner H (2008). Mutations and treatment outcome in cytogenetically normal acute myeloid leukemia. N Engl J Med.

[12] Arber DA, Stein AS, Carter NH, Ikle D, Forman SJ, Slovak ML (2003). Prognostic impact of acute myeloid leukemia classification. Importance of detection of recurring cytogenetic abnormalities and multilineage dysplasia on survival. Am J Clin Pathol.

[13] Haferlach T, Schoch C, Loffler H, Gassmann W, Kern W, Schnittger S, Fonatsch C, Ludwig WD, Wuchter C, Schlegelberger B, Staib P, Reichle A, Kubica U, Eimermacher H, Balleisen L, Grüneisen A, Haase D, Aul C, Karow J, Lengfelder E, Wörmann B, Heinecke A, Sauerland MC, Büchner T, Hiddemann W (2003). Morphologic dysplasia in de novo acute myeloid leukemia (AML) is related to unfavorable cytogenetics but has no independent prognostic relevance under the conditions of intensive induction therapy: results of a multiparameter analysis from the German AML Cooperative Group studies. J Clin Oncol.

[14] Pileri SA, Ascani S, Cox MC, Campidelli C, Bacci F, Piccioli M, Piccaluga PP, Agostinelli C, Asioli S, Novero D, Bisceglia M, Ponzoni M, Gentile A, Rinaldi P, Franco V, Vincelli D, Pileri A, Gasbarra R, Falini B, Zinzani PL, Baccarani M (2007). Myeloid sarcoma: clinico-pathologic, phenotypic and cytogenetic analysis of 92 adult patients. Leukemia.

[15] Matutes E, Morilla R, Farahat N, Carbonell F, Swansbury J, Dyer M, Catovsky D (1997). Definition of acute biphenotypic leukemia. Hematological.

[16] Hanson CA, Abaza M, Sheldon S, Ross CW, Schnitzer B, Stoolman LM (1993). Acute biphenotypic leukemia: immunophenotypic and cytogenetic analysis. Br J Haematol.

[17] Sulak LE, Clare CN, Morale BA, Hansen KL, Montiel MM (1990). Biphenotypic acute leukemia in adults. Am J Clin Pathol.

[18] Mirabelli P, Scalia G, Pascariello C, D'Alessio F, Mariotti E, Di Noto R, George TC, Kong R, Venkatachalam V, Basiji D, Del Vecchio L (2012). ImageStream promyelocytic leukemia protein immunolocalization: in search of promyelocytic leukemia cells. Cytometry.

[19] Shen Y, Zhu YM, Fan X, Shi JY, Wang QR, Yan XJ, Gu ZH, Wang YY, Chen B, Jiang CL, Yan H, Chen FF, Chen HM, Chen Z, Jin J, Chen SJ (2011). Gene mutation patterns and their prognostic impact in a cohort of 1185 patients with acute myeloid leukemia. Blood.

[20] Desangles F, Vilain E, Arborio M, De Revel T, Flandrin G (1995). t(15;17) hypergranular acute promyelocytic leukemia (M3) developing into a t(3;6) M3 without t(15;17) at relapse. Leuk Lymphoma.

[21] Hatzis T, Standen GR, Howell RT, Savill C, Wagstaff M, Scott GL (1995). Acute promyelocytic leukemia (M3): relapse with acute myeloblastic leukemia (M2) and dic(5;17)(q11;p11). Am J Hematol.

[22] Todisco E, Testi AM, Avvisati G, Moleti ML, Cedrone M, Cimino G, Mancini F, Amadori S, Mandelli F (1995). Therapy-related acute myelomonocytic leukemia following successful treatment for acute promyelocytic leukemia. Leukemia.

[23] Jubashi T, Nagai K, Miyazaki Y, Nakamura H, Matsuo T, Kuriyama K, Tomonaga M (1993). A unique case of acute promyelocytic leukemia (M3) developing into acute myeloblastic leukemia (M1) with t (7; 21) at relapse. Br J Haematol.

[24] Bonomi R, Giordano H, del Pilar MM, Bodega E, Landoni AI, Gallagher R, del Rosario UM (2000). Simultaneous PML/RARalpha and AML1/ETO expression with t(15;17) at onset and relapse with only t(8;21) in an acute promyelocytic leukemia patient. Cancer Genet Cytogenet.

[25] National Comprehensive Cancer Network Inc. NCCN: **Clinical Practice Guidelines in Oncology. Acute Myeloid Leukemia [EB/OL].** [www.nccn.org. 2011; version 2: 1-8]

[26] Nguyen DT, Diamond LW, Braylan RC (2003). Flow cytometry in hematopathology: a visual approach to data analysis and interpretation. Humana Press Inc.

[27] Béné MC, Porwit A (2012). Acute leukemias of ambiguous lineage. Semin Diagn Pathol.

[28] **Lithotripsy. Health and Public Policy Committee, American College of Physicians.** *Ann Intern Med* 1985; 103(4): 626-9. 4037564

[29] Stanley M (1985). Adult Leukemia 2. Boston. Martinus Nijhoff Publishers.

